# RC Medium-Rise Building Damage Sensitivity with SSI Effect

**DOI:** 10.3390/ma15051653

**Published:** 2022-02-23

**Authors:** Liga Gaile, Lasma Ratnika, Leonids Pakrastins

**Affiliations:** Faculty of Civil Engineering, Institute of Structural Engineering, Riga Technical University, LV-1048 Riga, Latvia; lasma.ratnika@rtu.lv (L.R.); leonids.pakrastins@rtu.lv (L.P.)

**Keywords:** structural health monitoring, soil–structure interaction, reinforced concrete buildings, natural frequencies, rocking frequencies, damage detection

## Abstract

Global vibration-based methods in the field of structural health monitoring are intended to capture structural stiffness changes of buildings or other civil engineering structures. Natural frequencies of buildings or bridges are commonly used parameters to monitor these stiffness changes. Therefore, it is essential to clarify the limit at which this method is no longer sensitive enough to be useful for structural health monitoring purposes. This paper numerically investigates the effect of structural damage and soil–structure interaction on cellular-type reinforced concrete buildings’ natural frequencies. These buildings are a common housing stock of Eastern Europe but are rarely investigated in this context. Comparisons with a reinforced concrete frame and infill structure building are made. Finite element models representing three structural system types of nine-story reinforced concrete buildings were used for the numerical simulations. Furthermore, a five-story finite element model was used for a damage sensitivity comparison. It is established that, for cellular-type structure buildings to detect damage comparable to that investigated in the paper, structural health (fixed base model frequency) should be monitored directly. Then, a statistical significance level for frequency changes of no more than 0.1% should be adopted. Conversely, the rocking frequency is a very sensitive parameter to monitor building base condition changes. These changes are often a cause of the cracking of building elements.

## 1. Introduction

The future development of smart buildings is unthinkable without introducing instrumented building monitoring systems that continuously monitor the structural performance of a building and provide early warning to building managers of signs of structural damage risks of degradation. Structural health monitoring is a scientific and engineering discipline that develops these types of solutions. Vibration-based methods for monitoring changes in the global stiffness of structures play an essential role in this field. This method is generally used for monitoring the technical condition of major high-rise buildings or bridges [1] with the development of suitable sensitive and accurate sensor systems [2]; it is also becoming possible to monitor low- and medium-rise structures, despite very low vibration amplitudes, which are on the order of 10^−6^ m/s^2^–10^−4^ m/s^2^ [3] and caused by ambient ground vibration.

Natural frequencies are the most commonly used parameters to identify building stiffness change using vibration-based methods [4]. The reason is the currently well-developed operational modal analysis (OMA) algorithms for the identification of these parameters from ambient vibrations [5] and developed methods for modeling environmental and operational variation effects (EOVs) on frequencies [6].

Therefore, a practical question becomes important: What kind of structural damage, and to what extent, is reflected in changes of natural frequencies of buildings? Most studies deal with analyzing individual elements in this context [7]. Some of the experimental studies on the impact of structural damage on whole-structure natural frequencies can be found in [8,9].

From these investigations, one of the crucial aspects to consider in medium-rise buildings is the soil–structure interaction effect (SSI), particularly the inertial SSI effect [10]. The effect is most pronounced when the building is located on weak or moderately weak soils [11]. In this case, the natural frequencies of the building are significantly reduced compared to a fixed base model. This poses a problem in identifying stiffness changes from changes in a modal parameter of the structure such as frequency since its change due to structural damage has a decreasing effect on the fundamental and other natural frequencies when the SSI effect is stronger [12].

One way to overcome this problem could be by performing a modal decomposition so that the shapes associated with the structural vibrations of the building are separated from the shape associated with the rocking motion. Then, the structural damage can be analyzed using the fixed foundation model, and the changes in the soil parameters (e.g., due to groundwater fluctuations [13]) can be analyzed using the rocking mode shape frequency.

**Figure 1:**
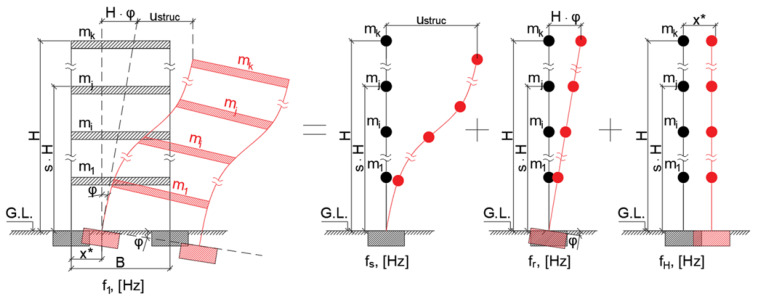
Multi-mass model scheme of building frame; f_1_—fundamental frequency, f_s_—frequency of shear frame action, f_r_—frequency of rocking action, f_h_—frequency of horizontal movement (not considered in this paper further), u_struc_—shear deformation of frame, H—building height, x*—horizontal displacement.

In this case, it is appropriate to model the building as a multi-mass model [14] (see Figure 1), so that low-amplitude ambient vibrations can be approximated as elastic deformations [15].

Depending on the geometry of the building, the shape of the lateral mode is as for a Euler–Bernoulli beam, pure shear beam, or somewhat in between. Hans and Boutin [16] developed a generic beam governed by a differential equation of the sixth degree to comprehend different types of buildings (see Equation (1)). The elastic parameters used in the equation are derived from elastic properties of the generic story under static shear or bending deformations. Depending on the structural behavior, the general model can degrade into a simpler model.

((E·I_µ_·E·I)/K)·U^(6)^ − (E·I_µ_ + E·I)·U^(4)^ − ((E·I)/K)·Λ·ω^2^·U^(2)^ + Λ·ω^2^·U = 0, (1)

where U^(x)^ is the horizontal displacement of the floors of the structure, K is the shear stiffness, EI is the global bending stiffness, EI_μ_ is the inner bending stiffness, Λ is the lineic density of structural material, and ω is the circular frequency.

However, there is no deformation of the structure itself in the rocking mode, but only a rotation of the foundation about a pivot point. The modal decomposition method was described in [17] where the coupling of all vibration modes with rocking action was considered.

This paper aims to numerically investigate the effect of structural damages on the natural-frequency changes of reinforced concrete buildings. Therefore, it contributes to understanding which kinds of medium-rise structures are appropriate for using the global vibration-based methods based on frequency sensitivity to the structural damage. In addition, curves are developed to estimate the percentage of frequency change using a fixed foundation model that is reflected in the percentage frequency change when the SSI effect through the rocking frequency is considered. This type of information can help at the planning stage, when the number and location of sensors and identification techniques need to be selected for structural monitoring, helping to understand whether the rocking motion of the building also needs to be identified separately. Particular attention is paid to cellular-type structures that are rarely investigated in this context. The term cellular means that the building structure is similar to stacked boxes, where each box can be considered as a cell, but the elements of the cells are structural elements.

It is established that, for cellular-type structure (nine-story) buildings, to detect damage comparable to that investigated in the paper, structural (fixed base model) frequency should be monitored directly. The statistical significance level for frequency changes should be adopted at no more than 0.1%.

Conversely, the rocking frequency is a very sensitive parameter to monitor building base condition changes that are often a cause of cracking of building elements and could be very useful.

## 2. Materials and Methods

### 2.1. Rocking and Structural Frequencies of Medium-Rise Reinforced Concrete Buildings

Full-scale experiments show that response due to ambient ground vibrations can be considered elastic for medium-rise buildings [18]. This means that the superposition principle applies, and the vibration modes of the structure can be decomposed as shown in Figure 1. Then, the fundamental frequency in the direction considered can be approximated by the following well-known relationship:

1/f_1_^2^ = 1/f_s_^2^ + 1/f_r_^2^, (2)

where f_1_ is the fundamental frequency in the direction considered, f_s_ is the structural fixed base model frequency in the direction considered, and f_r_ is the rocking frequency in the direction considered.

Typical fundamental frequencies are known from a number of experimental studies [19,20,21] and are given in Table 1.

The experimental frequencies shown in Table 1 include the frequency reduction due to the SSI effect. Therefore, by using the values in Table 1 as input parameter f_1_ in Equation (2), it is possible to determine theoretically which pairs of fundamental modal components—structural frequency f_s_ and rocking frequency f_r_—are even possible for a given range of buildings.

f_min_ ≤ (f_s_^2^·f_r_^2^/(f_s_^2^ + f_r_^2^))^1/2^ ≤ f_max_, where f_r_ ≤ f_s_. (3)

Research has shown [23] that the rocking frequency f_r_ is generally lower than structural frequency f_s_. If, due to the high stiffness of the structure, it acts more and more like a rigid body on the ground, then the rocking frequency effect increases [24]. Therefore, an additional constraint is introduced into Equation (3) whereby rocking frequency is less than or equal to the structural frequency. The system of inequalities is solved numerically by the simulation to obtain the possible combinations of modal components when f_s_ is taken in the range of 0.4–15 Hz.

Depending on the accuracy of the modal parameter identification, different statistical significance levels can be adopted for frequency changes due to structural damage, e.g., 0.1% (0.001), 0.5% (0.005), 1% (0.01), and 5% (0.05). It is, therefore, logical to consider structural frequency variations Δf_s_ due to damage at least in the range 0–5%. This variation can be expressed as a percentage difference between the reference-state structural frequency f_s_ and the structural frequency of damaged structure d_s_.

Δf_s_ = 100%·(f_s_ − d_s_)/f_s_, (4)

where Δf_s_ denotes structural frequency changes due to structural damage (%), f_s_ is the initial structural frequency (reference state), and d_s_ is the fundamental structural frequency of the damaged structure (modeled with fixed foundation).

The frequency change including the effects of SSI can be expressed as a percentage difference between the reference-state fundamental frequency f_1_ and the fundamental frequency of damaged structure d.

Δf_1_ = 100%·(f_1_ − d)/f_1_. (5)

In Equation (5), to find (f_1_ − d), a modification of Equation (2) can be used. Then, estimation of the frequency change (f_s_ − d_s_) using a fixed foundation model that is reflected in the frequency change (f_1_ − d) when the SSI effect through the rocking frequency is considered can be achieved using Equation (6).

(f_1_ − d) = ((f_s_ − d_s_)^2^·f_r_^2^/((f_s_ − d_s_)^2^ + f_r_^2^))^1/2^. (6)

### 2.2. Data for the Numerical Study

**Figure 2:**
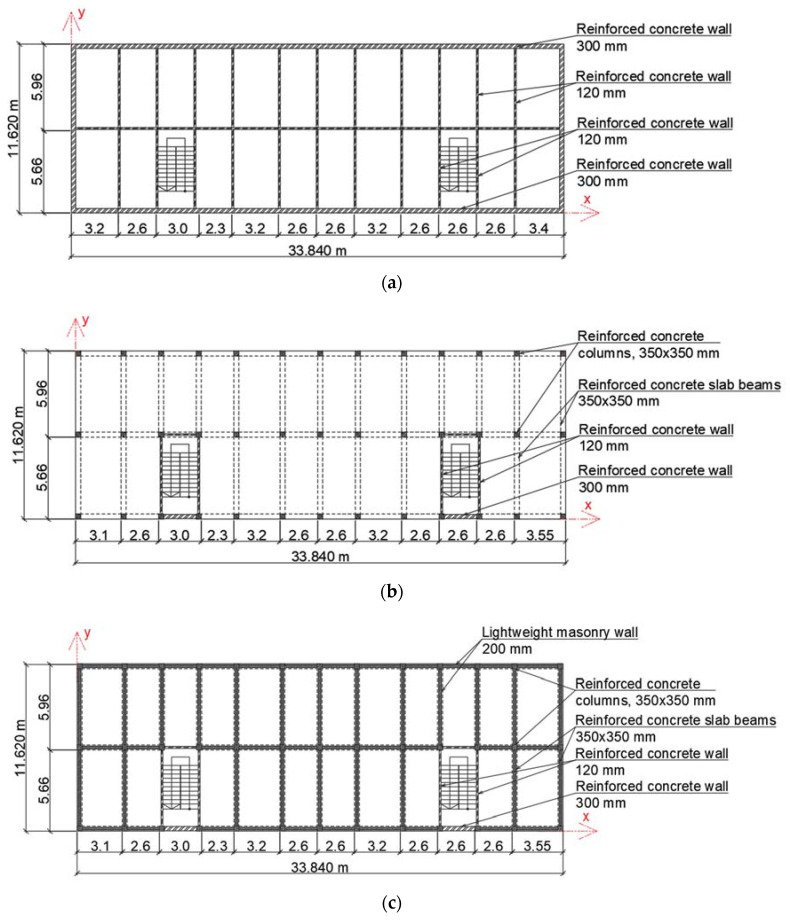
Principal schemes of investigated FEM reference models: (**a**) cellular structure, RC building (reference model #1); (**b**) frame structure, RC building (reference model #2); (**c**) frame structure with masonry infill walls, RC building (reference model #3).

In order to investigate the effect of the rocking frequency and structural changes on the natural frequency of three different building types, the following nine-story reference numerical models were created in the finite element model (FEM) software Dlubal RFEM using beam and plate finite elements (see Figure 2):

- cellular structure, reinforced concrete (RC) building (reference model #1);
- frame structure, RC building (reference model #2);
- frame structure with masonry infill walls, RC building (reference model #3).

The outer geometry was the same for all the models. The height of the buildings was 26.11 m, with plan dimensions of 11.620 × 33.84 m. The geometric dimensions of the individual elements are summarized in Table 2. Material constants for all reference models were taken as the same: modulus of elasticity 2490 kN/cm^2^, shear modulus 1037.50 kN/cm^2^, Poisson’s ratio 0.2, and specific weight 25 kN/m^3^. For infill walls, material constants were as follows: modulus of elasticity 1800 kN/cm^2^, shear modulus 750 kN/cm^2^, Poisson’s ratio 0.2, and specific weight 17.50 kN/m^3^.

Masonry infill was included in the appropriate manner in the finite element model according to [25]. Connections of the plate elements of the cellular precast building were modeled as a hinge joint between two surfaces. A fixed base model was considered for all reference buildings because the frequency sensitivity to damage considering the SSI effect could be later modified by the relationship obtained in this paper (see Section 3). The building structure itself was modeled elastically due to the fact that, under ambient vibrations, it has been experimentally proven that it behaves in this manner [18]. Natural frequencies of models were calculated using the Lanczos iterative method suitable for large models [26].

The structural damage to the building was assumed to be localized wall damage reflecting, for example, a newly formed doorway that was not foreseen in the original project and sometimes could be built illegally without the approval of the relevant authorities. Each model type had 11 interior wall planes in the longitudinal direction (*y*-direction). Structural damage was assumed for each interior wall plane until the symmetry axis of the building was reached. It is expressed as a percentage that was calculated as the ratio of the total wall plane area to the removed or damaged part of the wall area. For each plane, a damage percentage of 13% was assumed, and changes in the natural frequencies of the building were recorded (see also Section 3.2).

The global structural stiffness of the building described in Table 2 was calculated using FEM as the reference force applied to the building wall plane in *x*- or *y*-directions divided by the displacement of the building in *x*- or *y*-directions, respectively.

Using the three types of reference models and Equation (2), and supplementing these models with shallow foundations on three typical soils (the composition of which is summarized in Table 3, Table 4 and Table 5), the theoretical rocking frequency was also derived. Soil under the foundations was idealized as a homogeneous half-space with a linear–elastic, isotropic material [27]. The stiffness and damping properties of the soil were modeled using vertical and horizontal springs calculated for each soil type. The foundation parameters were calculated iteratively by means of a nonlinear method for every finite element depending on the loading from the building.

For investigations of the SSI effect on the building frequencies, nine FEM models were considered, whereas, for the damage sensitivity simulations, 192 FEM models were calculated.

## 3. Results

### 3.1. Modal Components of Medium-Rise RC Buildings

**Figure 3:**
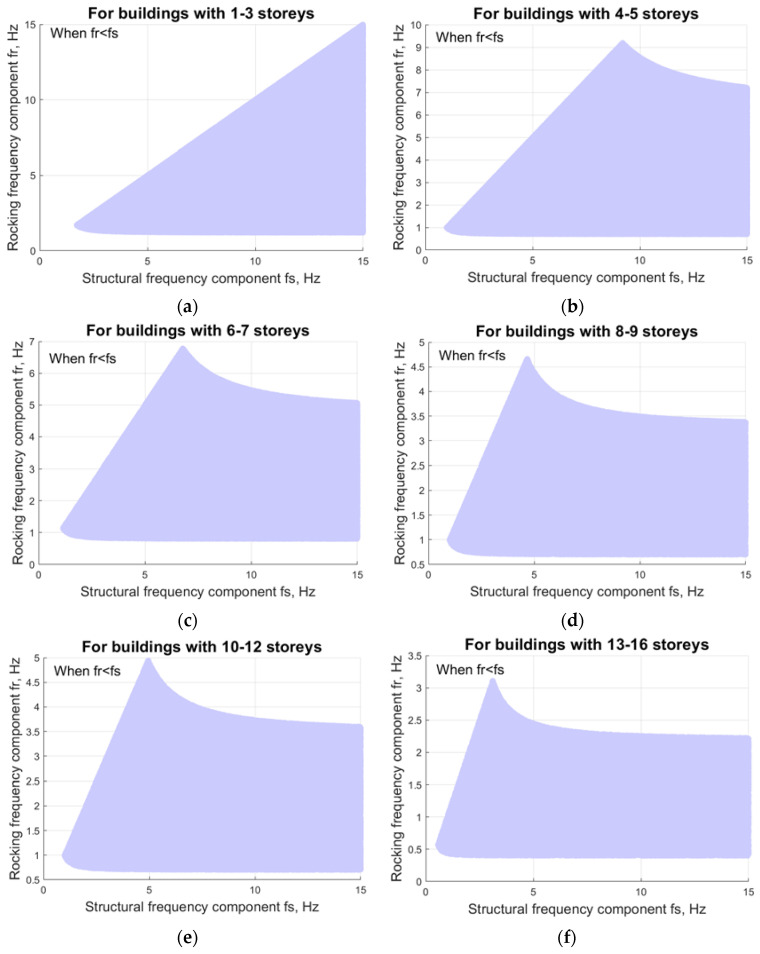
Modal component ranges of the first vibration mode for buildings: (**a**) 1–3 stories; (**b**) 4–5 stories; (**c**) 6–7 stories; (**d**) 8–9 stories; (**e**) 10–12 stories; (**f**) 13–16 stories.

Theoretically possible ranges for the modal components structural frequency fs and rocking frequency f_r_ of the first vibration mode for rigid buildings when fr < fs are summarized in Figure 3. These ranges were defined according to the methodology described in the previous section. These obtained ranges could be useful to assess experimental result credibility when performing modal parameter identification in practice.

**Figure 4:**
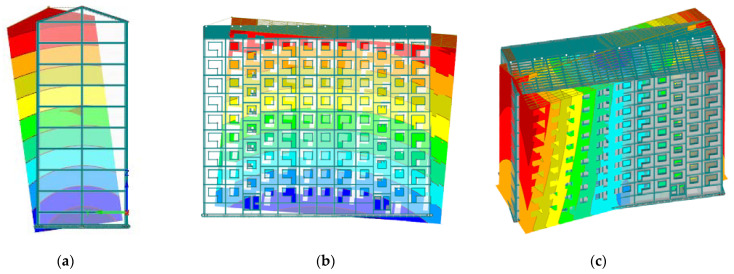
Fundamental vibration modes in each direction: (**a**) lateral mode; (**b**) longitudinal mode; (**c**) torsional mode.

Table 6 summarizes the results obtained from the finite element calculations, decomposing separately the contribution of each modal component using Equation (2) for the three vibration modes (Figure 4) and showing the ratio between structural mode and the mode with SSI effects including f_s_/f_1_.

This f_s_/f_1_ relationship reveals that the rocking frequency plays a very important role in the value of the lowest natural frequency, and its importance increases with the rigidity of the building on weaker soils. In none of the cases considered was f_r_ > f_s_ found; hence, the lowest ratio value obtained was 1.43. This is as it should be since the lowest possible value of the f_s_/f_1_ ratio is √2, which follows from Equation (2) in the case of f_s_ = f_r_.

A lower value denotes greater sensitivity of the fundamental frequency to the building damage scenario. For all soil types, the highest ratio f_s_/f_1_ values were found for the vibration modes in the direction of the smaller width of the building, which means that, if changes in soil parameters are to be monitored, it is valuable to look at the natural frequency of this vibration mode; however, if the structure itself is to be monitored, the torsional mode and lateral vibrational mode in the stiffer direction of the building should be looked at.

Table 7 analyzes the variation of rocking frequencies with soil type for buildings on shallow foundations.

It is clear that the soil type influences the rocking frequencies for all building types, especially for the first vibration mode in the narrowest building direction. For rigid buildings such as reference models #1 and #3, changes in soil composition have little effect on the rocking components of the torsional vibration mode.

According to the results in Table 7, the rocking frequency is a sensitive parameter for medium-height buildings to monitor changes in the building base condition. Changes in this parameter could promptly highlight future problems such as damage to the building structure due to uneven settlement, leading to cracking or undesirable displacements of horizontal members.

### 3.2. Effect of Structural Changes on the Natural Frequencies of RC Building

**Figure 5:**
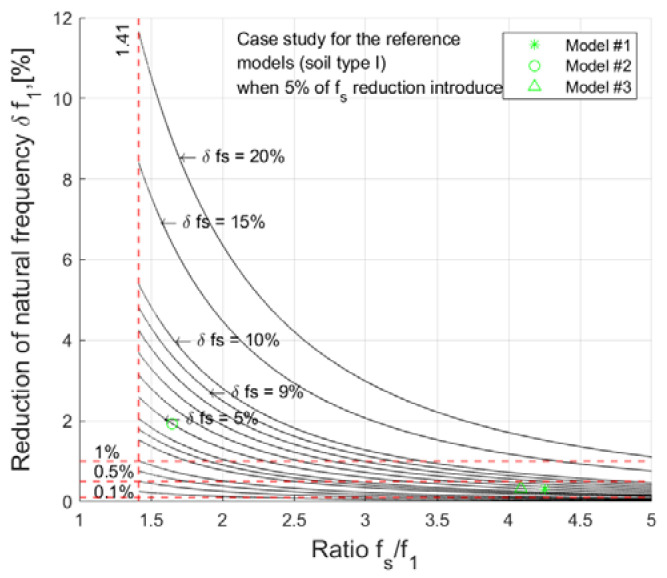
Sensitivity of natural frequency reduction to structural frequency f_s_ reduction.

Structural alternations of buildings change the structural frequency f_s_ (fixed foundation model). However, because rocking frequency considerably lowers the fundamental frequency of a building, these changes can have a minor effect on the fundamental frequency itself. To evaluate this effect, 10,000 simulations were performed with randomly selected values of rocking frequency f_r_ and structural frequency f_s_, as well as different levels of structural frequency changes δf_s_. Fundamental frequency f_1_ and change in fundamental frequency δf_1_ were calculated according to the methodology in Section 2.1, and the results are presented in Figure 5.

When analyzing the percentage change of the first vibration mode as a function of the building frequency ratio f_s_/f_1_ (under the condition that f_r_ ≤ f_s_), the curves tended asymptotically to a value of √2, with at most half of the structural frequency change being reflected in the first vibration mode (see Figure 5). If this ratio increases (i.e., the structure becomes stiffer relative to the deformability of the foundation), then the possibility of detecting structural damage from the frequency change of the first vibration mode decreases rapidly as the curve follows the power relationship. In Figure 5, the curves from lower to higher are plotted for the following f_s_ variations: 0.5%, 1%, 1.5%, 2%, 3%, 3.5%, 4%, 5%, 6%, 7%, 8%: 9%, 10%, 15%, and 20%.

Comparing the values in Table 7 with the graph in Figure 5, it follows that, for cellular or RC frame buildings with infill, where the change in the first frequency in each direction or in torsional frequency when structural damage changes the structural frequency f_s_ by less than 10%, the change in f_1_ is below the significance threshold of 0.5%. In this case, the solution is to identify and directly monitor the modal component f_s_.

**Figure 6:**
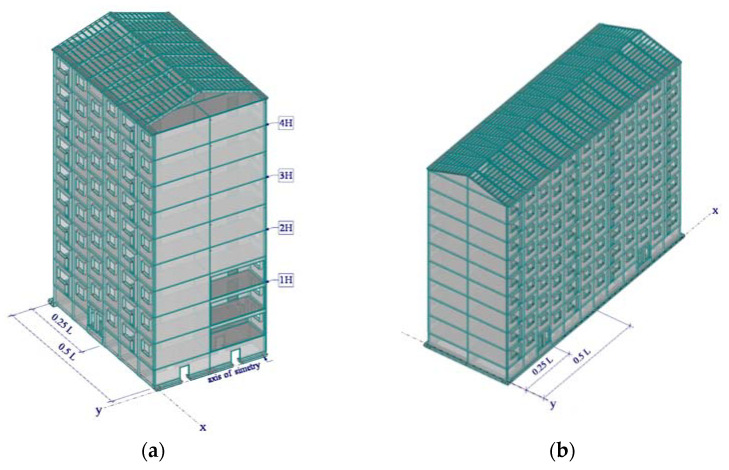
Damage locations for the reference model #1: (**a**) building section; (**b**) FE model of the building.

To understand how much damage to the building is required to produce sufficient changes in f_s_ for successful identification by operational modal analysis techniques, the most robust Model #1 was analyzed, successively taking out three panels at a time at each location (see Figure 6). In this case, a damage of 13% was calculated according to the methodology described in the previous section.

**Figure 7:**
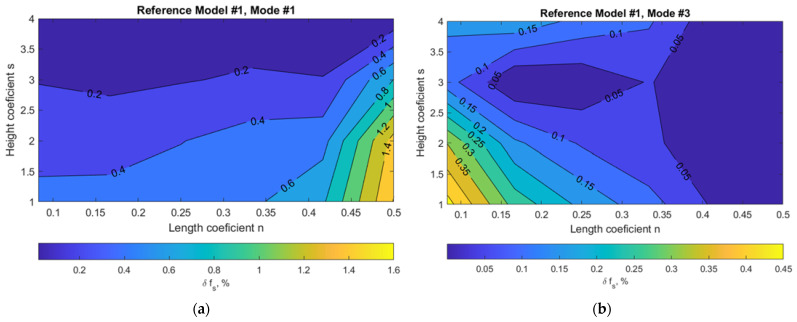
Variation of structural frequency f_s_ due to damage scenario for reference nine-story model #1: (**a**) mode #1 (lateral); (**b**) mode #3 (torsional).

**Figure 8:**
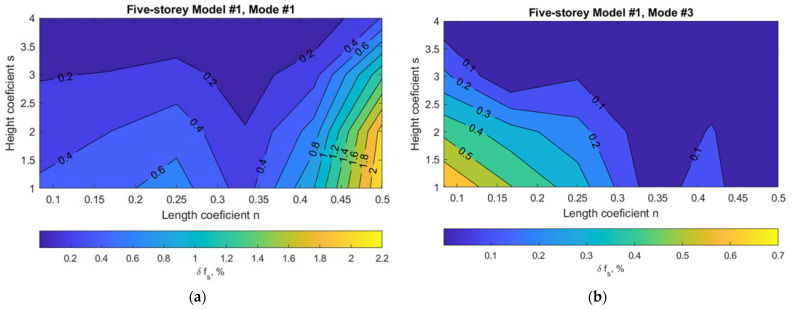
Variation of structural frequency f_s_ due to damage scenario for reference five-story Model #1: (**a**) mode #1 (lateral); (**b**) mode #3 (torsional).

The variation of structural frequency f_s_ due to the damage scenario for reference Model #1 depending on the damage location in the building plan or height is displayed in Figure 7 and Figure 8. The horizontal axis shows the dimension of the building in plan n × L, where 0.5 L corresponds to the axis of symmetry of the building in plan. The vertical axis corresponds to the building height s × H, which is up to the middle panel removed. (see Figure 6). To compare results of buildings with a different number of stories, a five-story building model was also chosen (with the same damage level of 13%).

As expected, the first vibration mode was the most sensitive for global damage identification purposes. The plot in Figure 8a (at distance 0.23 L) reveals that damage in walls around stiffness cores, e.g., staircases for lower buildings, influences a more fundamental frequency change of the building itself. Damage located in the middle of the building can be identified more easily when using the first lateral mode on the lower floors, while damage at the ends of the building can be identified more easily when using the torsional mode on the lower floors. This means that, for damage detection in building vertical elements that are located up to the midweight of the building, a statistical significance level of 0.25% (for frequency identification) can be adopted. However, the main conclusion is that statistical significance levels for frequency changes should be adopted at no more than 0.1% for such rigid buildings when aiming for damage detection in all vertical loadbearing building elements. When damage is even smaller than the damage considered in this study, monitoring of the structure itself using global vibration-based methods is not applicable for damage identification in this case.

## 4. Discussion

**Figure 9:**
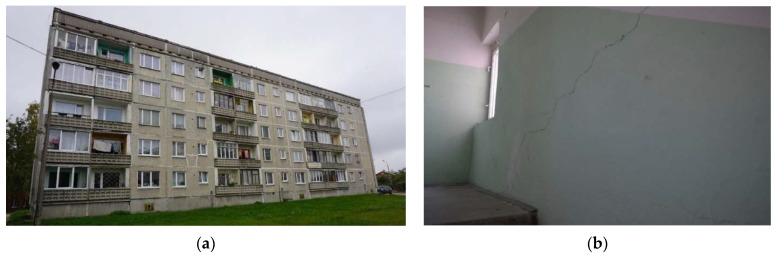
Cracks in residential buildings: (**a**) cracks in building façade panels; (**b**) cracks in building loadbearing walls.

The prototype for numerical models was a building widely used as a residential building in the former USSR (Figure 9a). The initiation of instrumented monitoring for such buildings is particularly relevant as they are approaching the end of their design life. Extending the lifetime of this type of housing stock would be a significant economic and ecological investment.

Frequently, damage or cracks in such buildings are caused by changes in ground conditions due to washouts, groundwater fluctuations, or nearby construction works, which are often difficult to prove for building owners. Therefore, a continuous monitoring system that identifies structural or base condition changes early could contribute to better management of those buildings. Examples of such cracks are shown in Figure 9b.

The results of the study presented in the previous section highlight the need for modal component detection by an operational modal analysis. These components are the structural frequency f_s_ corresponding to the fixed base condition and the rocking frequency f_r_ corresponding to the swaying motion of the building as a rigid body. There have been some attempts to do this on real buildings under ambient vibrations, e.g., [28,29]. However, the rocking motion for buildings has been mostly studied in the field of earthquake engineering as a technique to reduce and predict a seismic response [30].

In [31], the authors presented a linear finite element model (FEM) for damage identification of a 10-story reinforced concrete building using ambient vibration measurements. Damage was induced in the real building by experimentally removing six exterior infill walls. In that study, as in this one, it was found that the frequency reduction for mode #3 (torsional mode) was significantly smaller compared to that for modes #1 and #2. The reduction in the identified natural frequencies between the initial and the damage state for mode #1 and mode #2 was 6.25% and 7.19%; however, the reduction for mode #3 was only 0.6%. Compared to this study, when the maximum theoretical fundamental frequency change was only 1.4%, the experimental building had lower global stiffness and higher damage extent (six removed panels).

The parameter f_s_/f_1_ showing the ratio between structural mode frequency and structural frequency with SSI effects included denotes the building frequency sensitivity to structural damage events. This parameter obtained for a frame structure building is comparable to that found in similar studies (e.g., [12]). It is roughly 1.5 for soft soils. However, for cellular-type structures, it is more than 3, which is rarely found in the literature.

As for medium-height buildings such as cellular or frame buildings with masonry infills, the most sensitive modal component was found to be the rocking frequency f_r_. Thus, if modal updating is intended, the correct updating of the boundary conditions of the supports should receive the most attention. Rocking frequency is a parameter that is worthy of further study because of its sensitivity to change and because there is very little experimental research in this context. For example, it can be used to identify the causes of building damage such as cracking, as well as to develop an early warning system for building managers.

## 5. Conclusions

This study investigated the components of natural frequencies for medium-rise buildings, namely, rocking frequency and structural frequency. Charts of theoretically possible rocking and structural frequency pairs were presented for buildings with a different number of stories. These charts can be used for full-scale experimental investigation validation in practice, thus allowing a quick assessment of whether an experimentally identified structural or rocking frequency is realistic for the relevant type of building.

Simulations on three types of FEM models for nine-story buildings (cellular structure, moment-frame structure, and moment-frame structure with infills) revealed the sensitivity of rocking frequency to the changes in the foundation of medium-rise buildings. This especially holds for the cellular-type structural buildings or buildings with masonry infills. Therefore, it is suggested to consider rocking frequency as a damage-sensitive parameter in structural health monitoring applications for medium-rise buildings.

The effect of minor structural changes and their location (represented as widened door openings in three places of nine-story and five-story cellular-type buildings) was investigated by performing simulations on 192 FEM models. The main conclusions are as follows:

- For structural damage monitoring purposes of stiff medium-rise buildings (cellular type or moment-frame with infills), structural frequency (fixed base model) should be used instead of fundamental frequency (which includes the SSI effect); practically, this can be done by firstly identifying rocking frequency and fundamental frequency before calculating structural frequency;
- Statistical significance levels for structural frequency changes should be adopted at no more than 0.1% for such rigid buildings when aiming for damage detection in vertical loadbearing building elements. This requires experimental modal identification procedures performed with less than 0.1% uncertainty.

Further introduction of real damage for full-scale buildings and performing ambient vibration measurements on the updated reference models and damaged buildings are of interest to quantitatively compare the obtained results in this study.

## Figures and Tables

**Table 1:** Fundamental frequency ranges of buildings [22].

Number of Stories (Number of Assessed Buildings)	Typical Fundamental Frequency Range, Hz	Mean (Standard Deviation), Hz
1–3 (63)	1.2–12.5	6.1 (2.8)
4–5 (108)	0.7–6.5	3.8 (1.2)
6–7 (91)	0.8–4.8	2.8 (0.8)
8–9 (71)	0.7–3.3	2.1 (0.6)
10–12 (86)	0.7–3.5	1.9 (0.6)
13–16 (53)	0.4–2.2	1.3 (0.4)

**Table 2:** Structural geometry description of the numerical reference models.

Type of Element	Model #1	Model #2	Model #3
Main vertical elements	Shear walls with thickness 120 mm	Columns 350 × 350	Columns 350 × 350
Floor slabs	100 mm	100 mm	100 mm
Roof slab	100 mm	100 mm	100 mm
Infill walls	-	-	Lightweight masonry wall 200 mm
Foundation	Strip foundation 800 × 400 (h) mm	Strip foundation 800 × 400 (h) mm	Strip foundation 800 × 400 (h) mm
Global stiffness (direction *x*)	1,617,143 kN/m	27,610 kN/m	1,415,000 kN/m
Global stiffness (direction *y*)	1,197,857 kN/m	27,180 kN/m	1,081,935 kN/m
Total mass of building ^1^	8,386,020.66 kg	3,741,773.96 kg	8,308,993.32 kg

^1^ The combination of load used to calculate the natural frequencies was C = 1.0 × live load + 1.0 × self-weight of construction and layers.

**Table 3:** Soil materials of soil type I.

Soil Description	Soil Layer Parameters					
	Specific Weight		Modulus of Elasticity, MN/m^2^	Poisson’s Ratio ν	Thickness, m	Ordinate from Ground Level, m
	Unsaturated Weight γ, kN/m^3^	Saturated Weight γ_sat_, kN/m^3^				
Sand, closely graded	17.0	19.0	30.0	0.30	0.80	0.80
Sand	19.0	21.0	30.0	0.30	3.0	3.80
Dusty sand	18.0	20.3	18.0	0.30	4.0	7.80
Sand, gravelly sand	18.0	20.0	20.0	0.30	8.20	16.0

**Table 4:** Soil materials of soil type II.

Soil Description	Soil Layer Parameters					
	Specific Weight		Modulus of Elasticity, MN/m^2^	Poisson’s Ratio ν	Thickness, m	Ordinate from Ground Level, m
	Unsaturated Weight γ, kN/m^3^	Saturated Weight γ_sat_, kN/m^3^				
Sand, closely graded	17.0	19.0	30.0	0.30	0.80	0.80
Sand	19.0	21.0	30.0	0.30	3.0	3.80
Clay, low plasticity	19.0	19.5	2.50	0.42	4.50	8.30
Sand–clay mixture	18.0	19.0	10.0	0.35	7.70	16.0

**Table 5:** Soil materials of soil type III.

Soil Description	Soil Layer Parameters					
	Specific Weight		Modulus of Elasticity, MN/m^2^	Poisson’s Ratio ν	Thickness, m	Ordinate from Ground Level, m
	Unsaturated Weight γ, kN/m^3^	Saturated Weight γ_sat_, kN/m^3^				
Sand, closely graded	17.0	19.0	30.0	0.30	0.80	0.80
Sand	19.0	21.0	30.0	0.30	3.0	3.80
Peat	10.40	10.40	1.0	0.40	0.80	4.60
Dusty sand	18.0	20.3	18.0	0.30	3.7	8.30
Sand, gravelly sand	18.0	20.0	20.0	0.30	7.7	16.0

**Table 6:** Structural frequencies and their modal components for building reference models.

Type of Soil	Para-Meter ^2^	Mode # of Model #1			Mode # of Model #2			Mode # of Model #3		
		Lateral	Longitudinal	Torsional	Lateral	Longitudinal	Torsional	Lateral	Longitudinal	Torsional
I	f_1_	1.255	1.973	2.171	0.518	0.630	0.702	1.215	1.897	2.128
	f_s_	5.339	6.800	7.481	0.854	0.903	1.077	4.965	5.844	6.392
	f_r_	1.291	2.062	2.269	0.652	0.879	0.926	1.253	2.006	2.257
	f_s_/f_1_	4.25	3.45	3.45	1.65	1.43 ^3^	1.53	4.09	3.08	3.00
II	f_1_	0.867	1.628	2.164	0.433	0.603	0.670	0.838	1.573	2.113
	f_s_	5.339	6.800	7.481	0.854	0.903	1.077	4.965	5.844	6.392
	f_r_	0.879	1.677	2.261	0.518	0.810	0.856	0.850	1.633	2.239
	f_s_/f_1_	6.16	4.18	3.46	1.97	1.50	1.61	5.92	3.72	3.03
III	f_1_	1.025	1.796	2.166	0.476	0.615	0.684	0.993	1.73	2.118
	f_s_	5.339	6.800	7.481	0.854	0.903	1.077	4.965	5.844	6.392
	f_r_	1.044	1.862	2.263	0.573	0.840	0.886	1.013	1.811	2.245
	f_s_/f_1_	5.21	3.79	3.45	1.79	1.47	1.57	5.00	3.38	3.02

^2^ Parameter f_s_ does not depend on soil type; ^3^ the lowest ratio value obtained.

**Table 7:** Rocking frequency f_r_ according to soil type.

Type of Soil	Parameter ^1^	Mode # of Model #1			Mode # of Model #2			Mode # of Model #3		
		Lateral	Longitudinal	Torsional	Lateral	Longitudinal	Torsional	Lateral	Longitudinal	Torsional
I	f_r_	1.291	2.062	2.269	0.652	0.879	0.926	1.253	2.006	2.257
	Δf_r_	-	-	-	-	-	-	-	-	-
II	f_r_	0.879	1.677	2.261	0.518	0.810	0.856	0.850	1.633	2.239
	Δf_r_	46.87%	22.96%	0.35%	25.87%	8.52%	8.18%	47.4%	22.84%	0.80%
III	f_r_	1.044	1.862	2.263	0.573	0.840	0.886	1.013	1.811	2.245
	Δf_r_	23.66%	10.74%	0.27%	13.79%	4.64%	4.52%	23.69%	10.76%	0.55%

^1^ Where Δf_r_ is the percentage change in rocking frequency with respect to the building on type I soil for a given model and mode.

## Data Availability

Data are available on request to the authors.
